# Levels and in vitro functional effects of circulating anti-hinge antibodies in melanoma patients receiving the immune checkpoint inhibitor pembrolizumab

**DOI:** 10.1371/journal.pone.0290793

**Published:** 2023-09-15

**Authors:** Barry D. Hock, Liping Goddard, Sean A. MacPherson, Matthew Strother, David Gibbs, John F. Pearson, Judith L. McKenzie

**Affiliations:** 1 Haematology Research Group, Department of Pathology and Biomedical Science, University of Otago, Christchurch, Christchurch Hospital, Christchurch, New Zealand; 2 Haematology Department, Christchurch Hospital, Christchurch, New Zealand; 3 Canterbury Regional Cancer and Haematology Service, Christchurch, New Zealand; 4 Biostatistics and Computational Biology Unit, University of Otago, Christchurch, Christchurch, New Zealand; Kyoto University Graduate school, JAPAN

## Abstract

The efficacy of PD-1 monoclonals such as pembrolizumab can be modulated by the signals delivered via their Fc region. Tumour/inflammation associated proteases can generate F(ab’)_2_ fragments of therapeutic monoclonals, and subsequent recognition of F(ab’)_2_ epitopes by circulating anti-hinge antibodies (AHA) can then, potentially, link F(ab’)_2_ binding to the target antigen with novel Fc signalling. Although elevated in inflammatory diseases, AHA levels in cancer patients have not been investigated and functional studies utilising the full repertoire of AHA present in sera have been limited. AHA levels in pembrolizumab treated melanoma patients (n = 23) were therefore compared to those of normal donors and adalimumab treated patients. A subset of melanoma patients and the majority of adalimumab patients had elevated levels of AHA reactive with F(ab’)_2_ fragments of IgG_4_ anti-PD-1 monoclonals (nivolumab, pembrolizumab) and IgG_1_ therapeutic monoclonals (rituximab, adalimumab). Survival analysis was restricted by the small patient numbers but those melanoma patients with the highest levels (>75% percentile, n = 5) of pembrolizumab-F(ab’)_2_ reactive AHA had significantly better overall survival post pembrolizumab treatment (p = 0.039). *In vitro* functional studies demonstrated that the presence of AHA^+^ sera restored the neutrophil activating capacity of pembrolizumab to its F(ab’)_2_ fragment. Neither pembrolizumab nor its F(ab’)_2_ fragments can induce NK cell or complement dependent cytotoxicity (CDC). However, AHA^+^ sera in combination with pembrolizumab-F(ab’)_2_ provided Fc regions that could activate NK cells. The ability of AHA^+^ sera to restore CDC activity was more restricted and observed using only one pembrolizumab and one adalimumab patient serum in combination with rituximab- F(ab’)_2_. This study reports the presence of elevated AHA levels in pembrolizumab treated melanoma patients and highlight the potential for AHA to provide additional Fc signaling. The issue of whether tumour associated proteolysis of PD-1 mAbs and subsequent AHA recognition impacts on treatment efficacy requires further study.

## Introduction

Therapeutic antibodies that bind T cell expressed PD-1, and thereby block delivery of inhibitory signals, have revolutionised the treatment of malignancies such as melanoma [1]. The functional effects of antibodies result from not only antigen binding but also the subsequent, isotype dependent, signals delivered by their Fc regions [2, 3]. Previous studies have established the potential of Fc mediated signalling to modulate and, in some instances abrogate, the anti-tumour activity of anti-PD-1 mAbs [3–6]. Therefore, understanding the Fc mediated signals delivered as a consequence of therapeutic antibody binding, is critical to optimising their use.

The PD-1 specific therapeutics pembrolizumab and nivolumab were designed as IgG_4_ antibodies in order to avoid triggering Fc mediated death of the target T cell [2, 3]. However if these therapeutics are themselves recognised by antibodies of differing isotypes there is potential for the resulting complexes to trigger a different range of Fc mediated effects [7]. We have previously demonstrated that the binding of pembrolizumab specific anti-drug antibodies (ADA) increases Fc signalling over that induced by pembrolizumab alone [8].

An additional group of *in vivo* generated antibodies with the potential to recognise therapeutic monoclonals are anti-hinge antibodies (AHA) [9–16]. AHA do not recognise intact IgG but recognise epitopes that are exposed following proteolytic cleavage in the hinge region. The resulting single cleaved IgG and/or F(ab)_2_ fragments retain bivalent antigen binding but have no Fc mediated effector functions [11, 17–20]. Proteases capable of lower hinge cleavage are found within inflammatory and/or tumour microenvironments [11, 19] and increased tissue levels of cleaved IgG have been associated with poor outcomes in breast cancer and arthritis [17, 18, 21, 22].

AHA are widely detected in healthy donors and found at elevated levels in patients with inflammatory diseases [9, 10, 12–14]. The binding of AHA to cleaved IgG can provide surrogate Fc effector functions [9, 13, 15, 16, 20] and, depending on their isotype(s), may provide additional Fc mediated effects [13, 23]. *In vivo* studies using murine tumour models have confirmed that AHA can induce anti-tumour responses that reverse the effects of IgG cleavage [22, 24].

The relative levels of AHA in melanoma patients is currently unknown. A number of matrix metalloproteases (MMP) involved in melanoma progression such as MMP-3, MMP-7 and MMP-12, are able to cleave IgG within the lower hinge region [25–28]. Therapeutic PD-1 antibodies have been shown to be sensitive to hinge region cleavage at least *in vitro* [19]. This, together with the presence of infused therapeutic PD-1 antibodies at melanoma tumour sites [29] raises the possibility that cleaved forms of PD-1 antibodies may be generated *in vivo* and, following recognition by AHA, modulate the functional outcomes of anti-PD-1 therapy [3–6, 9, 15, 16, 20, 23].

Although the presence of elevated AHA levels in patients with inflammatory conditions is well established, much remains unclear concerning the presence and function of AHA. There is no published data available regarding AHA levels in cancer patients or indeed patients receiving antibody therapies of any type. To date, studies analysing reactivity with cleaved IgG_4_ have utilised two antibodies that lack the hinge stabilising mutation present in IgG4 anti-PD-1 monoclonals such as pembrolizumab [12–14, 30]. Non-stabilised IgG_4_ and pembrolizumab differ markedly with respect to the conformation of their cleaved fragments [31]. As AHA can recognise conformational epitopes, the findings reported using cleaved non-stabilised antibodies are not necessarily representative of reactivity with cleaved pembrolizumab [15, 30]. Functional studies using AHA have focused almost entirely on their ability to restore function to cleaved IgG_1_ and the limited analysis performed using cleaved IgG_4_ as a target, utilised non-hinge stabilised IgG_4_ [13, 23]. Additionally, functional studies utilising AHA have, to date, been performed almost entirely with monoclonal AHA or AHA purified from pooled serum, which preferentially enriches those of highest affinity. Present data indicates the AHA repertoire differs between individuals and is comprised of multiple isotypes, specificities and affinities [14, 15]. Given that the functional effect of specific antibodies in serum can be modulated by the presence of other antibodies directed against the same antigen [32, 33] it is unclear what level of AHA mediated functional effects will be observed in the more physiological setting of individual sera that contains a full repertoire of AHA.

In this study, we compared the levels of AHA in melanoma patients receiving pembrolizumab with those in both healthy donors and patients with inflammatory diseases receiving the TNF specific antibody adalimumab. In addition the potential functional effects of AHA present in sera from individual melanoma patients was analysed.

## Materials and methods

### Cell and serum preparation

Blood was collected from normal donors (ND) and patients who had provided written consent. Ethical approval was obtained from Health and Disability Ethics Committees, New Zealand.

Patients were recruited in the period 2017–2018 and the authors had access to identifying information. Serum was obtained by incubation (2hr/RT) then centrifugation (1200xg/20min) of blood and then stored frozen. Three sets of serum were prepared. The first was from ND. The second set was from patients receiving adalimumab as treatment for either inflammatory bowel disease or arthritis and was collected immediately prior to an infusion. The third set was collected as part of a previous study [8] from patients receiving pembrolizumab as treatment for stage IV melanoma. Samples were collected immediately pre (trough) each 3-weekly cycle of infusion with patients first entering the study at different cycles (1–9) All patient serums were negative in assays testing for the presence of anti-drug antibodies [8, 34].

Peripheral blood mononuclear cells (PBMC) and granulocytes were obtained by centrifugation of blood over Ficoll/Paque (Amersham Pharmacia Biotech, Uppsala, Sweden). Contaminating red blood cells in granulocyte preparations were removed by NH_4_Cl lysis. As described previously [8] NK cells were enriched (40–55% CD56^+^) from PBMC by negative selection using CD14, CD19 and CD3 mAb, in combination with Goat–anti-mouse-IgG coated Dynabeads (Invitrogen). A PD-1 expressing Jurkat cell line was a kind gift from Professor Alexander McLellan, University of Otago, New Zealand. In brief the cell line was generated using the full length PD-1 gene amplified from the cDNA of PMA+ionomycin activated human splenocytes and subsequently cloned into the Sfil sites of pSB-bi-G418. The media used in cell based experiments was RPMI (Sigma, St Louis, MO) supplemented with 10% heat inactivated fetal calf serum (FCS, Invitrogen, Auckland, New Zealand) unless otherwise indicated.

### Therapeutic monoclonals and flow cytometry

Stocks of therapeutic monoclonals were obtained from either injection vials of rituximab (MabThera, Roche), nivolumab (OPDIVO, Bristol-Myers Squibb) and pembrolizumab (Keytruda, Merck) or pre-filled injection pens of adalimumab (Abbott Laboratories). CD25-PE, CD16-PE and CD56-APC were obtained from BD Biosciences. Flow cytometric analysis was performed on a Beckman Coulter FC500 MPL flow cytometer, and results are expressed as mean fluorescence intensity (MFI).

### Generation of F(ab’)_2_ fragments

F(ab’)_2_ fragments were generated using a F(ab’)_2_ preparation kit (Pierce) that utilised immobilized pepsin for digestion in combination with Protein A removal of unbound antibody. Optimal digestion buffers and 37°C incubation times differed between antibodies and were (i) 4h in 20mM sodium acetate (pH 4.4) for pembrolizumab and rituximab (ii) 1h in 20mM sodium acetate (pH 4.4) for nivolumab and (iii) 16h in 0.1M sodium Citrate (pH 3.5) for adalimumab. Following removal of low mw fragments by ultrafiltration using a centrifugal concentrator with 30 kDa MWCO (Sartorious), the purity of F(ab’)_2_ fragments was analysed by western blotting and ELISA. All fragments had a single band at approximately 100 kDa following SDS-PAGE performed as described previously [35]. All purified F(ab’)_2_ when coated (1 μg/ml) onto ELISA plates were strongly stained by a Goat Anti-Human IgG reagent reactive with both heavy and light chains (GaHIg-H&L, Sigma) but were not recognised by a Fc specific Goat anti-Human IgG reagent (bio-GAH-Fc, Sigma). The antigen binding capacity of purified F(ab’)_2_ compared to the respective parent antibody was confirmed by either capture ELISA using recombinant TNF or PD-1 (R&D systems) as the target or in the case of rituximab by demonstrating the ability to block binding of a CD20-PE antibody to the CD20^+^ cell line Raji.

### AHA ELISA’s

AHA levels were analysed by ELISA using a modification of our previously described ELISA methodology [34]. Briefly, ELISA plates (Costar) were coated (16h, 4°C) with F(ab’)_2_ (100 μl/well at 0.5 μg/ml) then following washing (0.1% Tween 20/PBS) blocked with 1% milk powder (MP). Serum samples diluted 1 in 200 in buffer (1% MP, 20 mM EDTA, 0.1% Tween 20) were added to the wells. Following incubation (90 min, 37°C) plates were washed and bound AHA detected by incubation (1h, 37°C) with goat anti-human IgG biotin [Fc-specific F(ab´)_2_, Sigma] diluted in 1% goat serum and then, following washing, further incubation (30 min, 37°C) with streptavidin–horseradish peroxidase enzyme (Dako, Glostrup, Denmark) diluted 1 in 7000 with 0.2% bovine serum albumin/PBS. After washing and addition of TMB+ substrate (Dako) the reaction was stopped after 6 minutes by acidification (3 mol/L H_2_SO_4_), and the absorbance was read at 450 nm. Absorbance’s for all F(ab’)_2_ fragments were normalised against the signal generated by a AHA^+^ control serum reacting with pembrolizumab- F(ab’)_2_ on the same plate (mean O.D ±SD = 0.99±0.12). Inhibition assays were performed using diluted serum samples pre-incubated (30 min) with either nil, antibody or F(ab´)_2_ at 20 μg/ml. Depletion experiments were performed using serum that were pre-incubated (1hr) with either nil or a PD-1 expressing cell line that was pre labelled with either pembrolizumab or pembrolizumab-F(ab’)_2_. Following incubation cells were removed by centrifugation and the supernatant analysed. In some experiments plates were coated (2h, 37°C) with 2 μg/ml recombinant PD-1 (R&D systems) and then following washing and blocking of plates as above wells were incubated (16h, 4°C) with pembrolizumab-F(ab’)_2_ (0.5 μg/ml in 1%MP) prior to washing, incubation with serum and detection of bound AHA as detailed above.

### NK activation assay

NK activation was analysed using a modification of our previously described method [8]. ELISA plates were pre-coated (2h, 37°C) with/without IgG or F(ab´)_2_ at 10 μg/ml. Following washing, wells were incubated (30 min) with 100μl patient or control serum (diluted 1 in 50 in media) prior to addition of 100 μl enriched NK cells (2.5x 10^5^) or PBMC (3x10^5^) in media containing 50 ng/ml IL-2 (R&D Systems). Following incubation (20h/37°C) the enriched NK cells were immune-labelled with CD16-PE and CD56-APC and analysed by flow cytometry. Cytotoxicity of cultured PBMC was assessed in a 4h killing assay using K562 as a target at a 5:1 effector:target ratio. The K562 was labelled with carboxyfluorescein diacetate, succinimidyl ester (CFSE; Invitrogen, Auckland, New Zealand) and cultured overnight prior to use and Propidium iodide (PI, Sigma) labelling in combination with flow cytometry was used to determine the proportion of target death (PI^+^CFSE^+^/ Total CFSE^+^).

### ROS production by granulocytes

ROS production was analysed using a modification of our previously described method [8]. ELISA plates were pre-coated (2h, 37°C) with/without IgG or F(ab´)_2_ at 10 μg/ml. Following washing and blocking of plates with 1%MP/PBS (1h, 37°C), wells were incubated (1h, 37°C) with 100μl patient or control serum (diluted 1 in 50 in media) prior to washing.

Granulocytes (1x10^6^/ml) were incubated (15min/37°C) with ROS indicator Dihydrorhodamine 123 (Invitrogen, 5uM) then 200ul added per well of an ELISA plate either uncoated or coated as described above. Following incubation (1h/37°C), the MFI of green fluorescent rhodamine 123 was determined by flow cytometry.

### Complement dependent cytotoxicity (CDC) assay

Target cells (1x10^5^) in 25μl media were added to wells of a 96 well U bottom plate. A further 25μl of media supplemented with either nil, antibody or F(ab’)_2_ fragments (20 μg/ml) was added in combination with nil or 2 μl serum from either ND or patients. Following incubation (2h, RT) 100μl of rabbit serum (16% in HBSS) was added and plates incubated a further 40 minutes (37°C) prior to addition of PI and determination of the proportion of PI^+^ non-viable cells by flow cytometry. Each ND/patient serum sample was incubated in both the presence and absence of antibody/ F(ab’)_2_ and the difference between the treatments provided the measure of specific lysis.

### Statistics

AHA levels in ND and patients had non-normal distributions and were therefore compared by analysis of log transformed raw data using ANOVA in combination with Dunnett’s post hoc test. Comparisons of different treatments were performed by analysis of raw data using repeated measures ANOVA in combination with a Holm-Šídák’s multiple comparisons test. Asterisks indicate significance _^*^P*<*0.05; ^**^P*<*0.01; ^***^P*<*0.001; ^****^P*<*0.0001. The relationship between continuous variables was evaluated using Spearmans rank correlation coefficient. Survival distributions were plotted using Kaplan-Meier plots and compared by the log rank test. Analysis of scaled schoenfeld residuals was used to check the validity of the assumption of proportion hazards. Evaluation of the optimal cut-point for AHA levels with respect to overall survival was performed using maximally selected rank statistics as implemented in the maxstat R package (https://CRAN.R-project.org/package=maxstat). All other analysis and graphing was performed using GraphPad Prism version 9.4.0 for Windows (GraphPad Software, La Jolla, California USA).

## Results

### Serum levels of AHA

**Fig 1:**
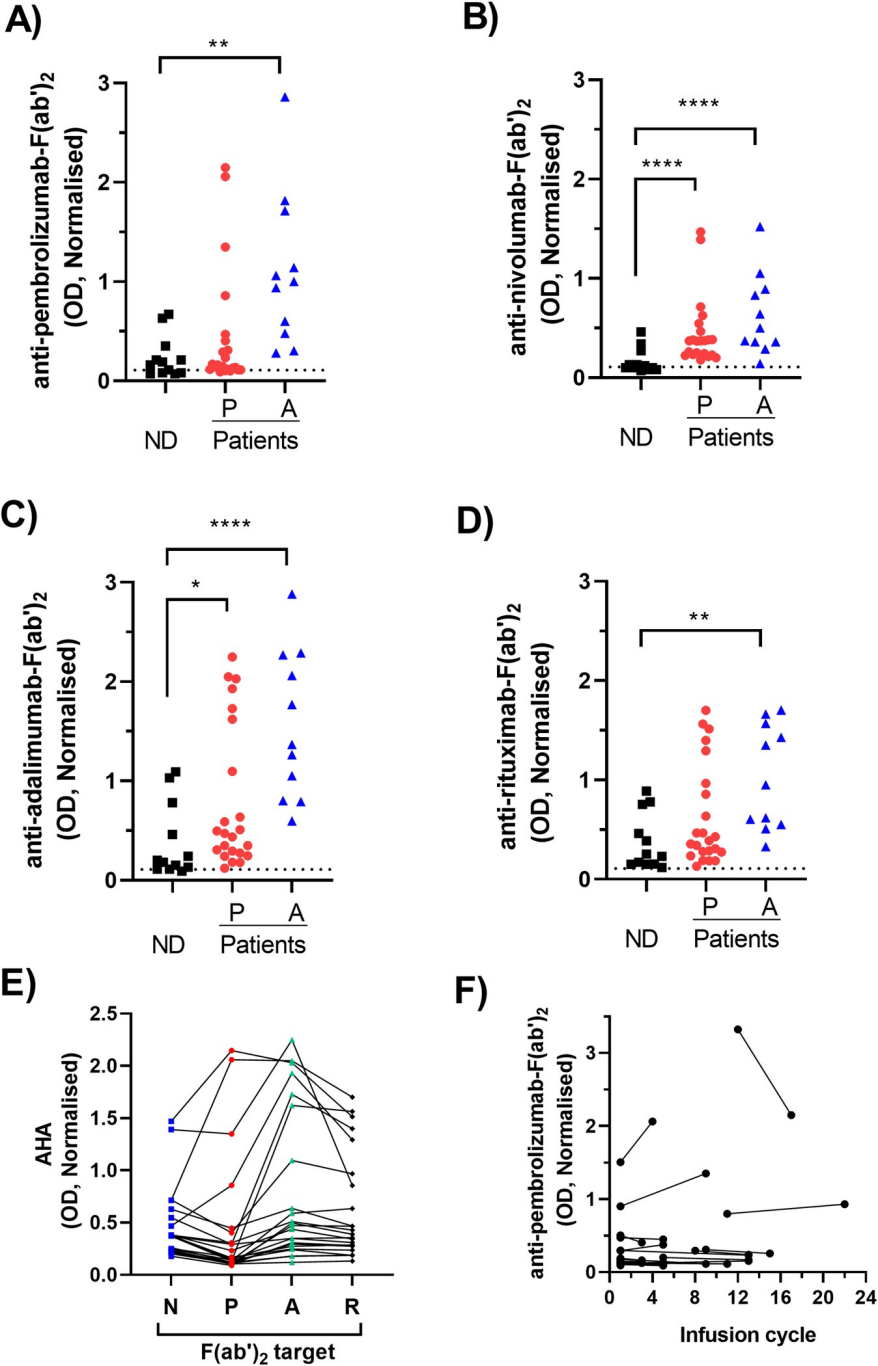
Comparison of AHA levels in patients and controls. Serum from normal donors [ND], adalimumab treated patients [A] and pembrolizumab treated patients [P] were analysed by ELISA for reactivity against F(ab’)_2_ fragments. All O.D’s measured by ELISA were normalised against the signal generated by a control serum reacting with pembrolizumab- F(ab’)_2_ on the same plate (mean O.D ±SD = 0.99±0.12). (A-D) Normalised AHA data are shown as a scatter plot of reactivity with the F(ab’)_2_ fragments of (A) pembrolizumab (B) nivolumab (C) adalimumab and (D) rituximab and the dotted line indicates the cut-point for positivity. Asterisks indicate a significant difference from ND levels following analysis of log transformed raw data by ANOVA in combination with Dunnett’s post hoc test (E) the F(ab’)_2_ reactivity of each pembrolizumab treated patient serum are shown as a line graph. Each line represents an individual serum and the columns correspond to the different F(ab’)_2_ targets nivolumab-F(ab’)_2_ [N], pembrolizumab-F(ab’)_2_ [P], adalimumab-F(ab’)_2_ [A] and rituximab-F(ab’)_2_ [R] (F) Levels of pembrolizumab- F(ab’)_2_ reactive AHA detected in serum collected from each pembrolizumab treated patient at two time points corresponding to the indicated treatment infusion cycle. Serum was collected immediately prior to infusion and therefore cycle 1 samples represent pre-treatment. Data from each individual patient are shown as a line linking two samples, with the later sample corresponding to that used throughout the rest of the study.

Levels of serum AHA reactive with F(ab’)_2_ fragments were analysed using a direct ELISA which specifically detects IgG-Fc (Fig 1A–1D). The F(ab’)_2_ fragments were prepared from hinge modified IgG_4_ (pembrolizumab, nivolumab) and IgG_1_ (adalimumab, rituximab) monoclonals which are widely used therapeutically. Sera from melanoma patients receiving pembrolizumab were the primary test group. Sera obtained from normal donors (ND) provided a baseline control. As AHA levels have previously only been studied in patients with inflammatory diseases, serum from inflammatory disease patients treated with adalimumab were included as a comparator group.

Only low levels of non-specific signal was observed in the absence of either plate bound F(ab’)_2_ or serum samples and this signal was used to define the cut-point for positivity (mean+3xSD = 0.109). AHA levels in normal donor serum (ND, n = 12) were predominantly low against all F(ab’)_2_ preparations with the majority having levels less than twice the positivity cut-point (defined as AHA^Low^). Relative to ND, serum from adalimumab treated patients (n = 11) had significantly higher levels of AHA reactive with each F(ab’)_2_ preparation.

The overall levels of pembrolizumab-F(ab’)_2_ reactive AHA in pembrolizumab treated patient samples (n = 23) were not significantly different from those of ND (Fig 1A). However, a subset (4/23) had levels higher than the upper level observed in normal donors (defined as AHA^High^). A similar pattern of reactivity was observed using nivolumab- F(ab’)_2_ as the target antigen (Fig 1B). A higher proportion of samples from pembrolizumab treated patients (6/23) were AHA^High^ in assays using F(ab’)_2_ derived from IgG_1_ antibodies (adalimumab, rituximab) rather than IgG_4_- F(ab’)_2_ as the target antigen. The highest levels of AHA were detected using adalimumab- F(ab’)_2_ as the target antigen and overall both pembrolizumab and adalimumab treated patients had levels significantly higher than those of normal donors (Fig 1C and 1D).

Further analysis of the data from pembrolizumab treated patients was performed to determine whether individual sera had elevated reactivity with only a single or multiple F(ab’)_2_ targets (Fig 1E). The comparison of results from each individual serum demonstrated that all four sera with elevated reactivity against pembrolizumab- F(ab’)_2_ also had elevated reactivity with adalimumab- F(ab’)_2_ and rituximab- F(ab’)_2_. However only two of these samples also had elevated reactivity with nivolumab-F(ab’)_2_. An additional two samples with lower pembrolizumab- F(ab’)_2_ reactivity did have elevated reactivity with both adalimumab- F(ab’)_2_ and rituximab- F(ab’)_2_. In contrast, comparison of the AHA reactivity of serum from each individual adalimumab treated patient showed each serum had the same relative level of reactivity with all the F(ab’)_2_ fragments. A single adalimumab treated patient had the highest level of reactivity against all F(ab’)_2_ fragments and was used as a positive control (AHA^+Con^) in subsequent experiments.

Serum samples were collected from the pembrolizumab treated patients at two or more different cycles of their treatment. The final collected sample was the one used in the analysis detailed in this study. Comparison of AHA levels in the first versus final collected sample demonstrated that levels stayed relatively high or low over the course of multiple treatment cycles (Fig 1F). Serum was collected immediately prior to each infusion and therefore samples collected at cycle 1 correspond to pre-treatment samples. Elevated AHA levels were observed in 2 of the 17 samples collected pre-treatment.

**Fig 2:**
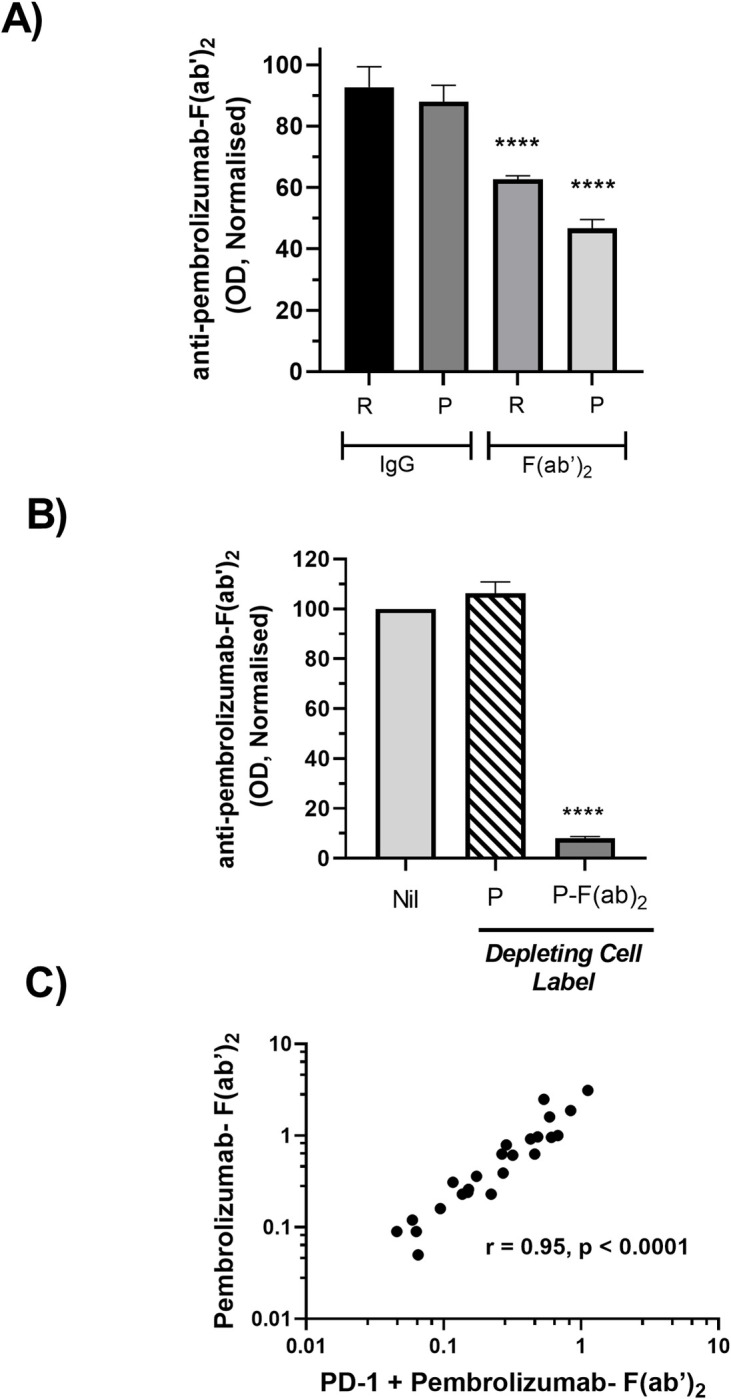
Specificity of AHA detection. (A) Serum containing AHA (n = 3) were divided into aliquots and spiked with either nil, rituximab, rituximab- F(ab’)_2_, pembrolizumab or pembrolizumab- F(ab’)_2_ prior to ELISA based determination of AHA reactivity with solid phase pembrolizumab-F(ab’)_2_. The AHA levels detected in each serum aliquot were normalised relative to the level in the corresponding nil treated aliquot (defined as 100%) and pooled data are shown as mean ± SEM. The serums analysed were obtained from a ND, a pembrolizumab treated patient and an adalimumab treated patient that had AHA OD of 0.6, 0.8 and 1 respectively. (B) Serum containing AHA (n = 4) were divided into aliquots and either nil treated or depleted by incubation with a PD-1+cell line that had been labelled with either pembrolizumab or pembrolizumab- F(ab’)_2_. AHA reactivity with solid phase pembrolizumab-F(ab’)_2_ was then determined by ELISA and the levels in each serum aliquot were normalised relative to the level in the corresponding nil treated aliquot (defined as 100%) and pooled data are shown as mean ± SEM. The serums analysed all had elevated AHA levels and were obtained from AHA^+Con^, a pembrolizumab treated patient and 2 adalimumab treated patients (C) AHA reactivity with pembrolizumab- F(ab’)_2_ that has bound to its target antigen PD-1. AHA binding was detected by ELISA and results normalised as described in Fig 1. Data are shown as a scatterplot of reactivity with PD-1 bound pembrolizumab-F(ab)2 versus reactivity with solid phase pembrolizumab—F(ab’)_2_ for each individual serum sample. Serum analysed were from ND and adalimumab treated patients and the respective Spearman rank correlation coefficient is indicated. Asterisks in A and B indicate treatments significantly different from nil following analysis of raw data by repeated measures ANOVA.

The serum samples from adalimumab and pembrolizumab treated patients all contained the respective drug at relatively high levels (10–40 μg/ml). The detection of AHA reactive with the F(ab’)_2_ fragment of the same drug in these samples provides strong evidence that the AHA recognise a cryptic epitope revealed only following hinge cleavage. The specificity of the AHA ELISA was further investigated by inhibition experiments using control and patient serum samples that had elevated reactivity with pembrolizumab- F(ab’)_2_ (Fig 2A). The addition of excess pembrolizumab or rituximab to serum samples did not inhibit the signal whilst addition of the corresponding- F(ab’)_2_ fragments resulted in a significant but incomplete reduction of the ELISA signal. It has been reported that anti-hinge antibodies recognise both linear and conformational epitopes. Additionally binding of antibodies to either antigen or solid phases can reveal cryptic epitopes not accessible on the corresponding soluble antibody. This raises the possibility that the ELISA detects AHA reactive not only with soluble F(ab’)_2_ fragments but also cryptic epitopes on antigen bound F(ab’)_2_ fragments. Therefore, to further confirm specificity, depletion experiments were performed using antigen bound forms of pembrolizumab and pembrolizumab- F(ab’)_2._ In these experiments a PD-1^+^ cell line was labelled with either pembrolizumab or pembrolizumab- F(ab’)_2_ then used to deplete AHA^+^ sera prior to ELISA analysis (Fig 2B). Depletion with pembrolizumab labelled cells did not reduce the ELISA signal whereas depletion with pembrolizumab- F(ab’)_2_ labelled cells resulted in almost complete reduction of the ELISA signal. These results provide strong evidence that the ELISA specifically detects AHA reactive with the epitopes accessible on antigen bound forms of F(ab’)_2_ fragments and suggests at least some of these epitopes are less accessible on the soluble forms of F(ab’)_2_ fragments. Additionally the lack of signal depletion using pembrolizumab labelled cells provides strong evidence that Fc reactive antibodies such as rheumatoid factors (RF) do not contribute to the ELISA signal.

The ability of AHA to recognise pembrolizumab- F(ab’)_2_ bound to its target antigen PD-1 was also analysed by ELISA. Analysis was restricted to ND and adalimumab treated-patient sera as those samples did not contain circulating pembrolizumab. Reactivity with PD-1 bound pembrolizumab- F(ab’)_2_ was strongly correlated with that observed using directly coated pembrolizumab- F(ab’)_2_ (Fig 2C).

It has been reported that a subset of IgG_4_-F(ab’)_2_ reactive AHA have an IgA isotype [14]. However none of the analysed serums showed reactivity with pembrolizumab- F(ab’)_2_ in a direct ELISA using IgA specific detection (Figure A in S1 Fig). A similar analysis using an IgM specific detection reagent did however detect low levels of IgM-AHA in three patient serums (Figure B in S1 Fig). Two of these serums were from adalimumab patients and also had moderate levels of IgG-AHA detected. The serum with the highest levels of IgM-AHA was from a melanoma patient (AHA^IgM^) and lacked detectable IgG-AHA. The potential presence of RF was also assessed. RF are antibodies that recognise the Fc region of other antibodies >90% of RF positive sera contain IgM-RF [36, 37]. All sera were therefore screened for reactivity with non-cleaved pembrolizumab using IgM specific detection (Figure C in S1 Fig). Only a single serum gave a positive signal indicating a low prevalence of RF in these samples.

### Association between AHA levels and overall survival

**Fig 3:**
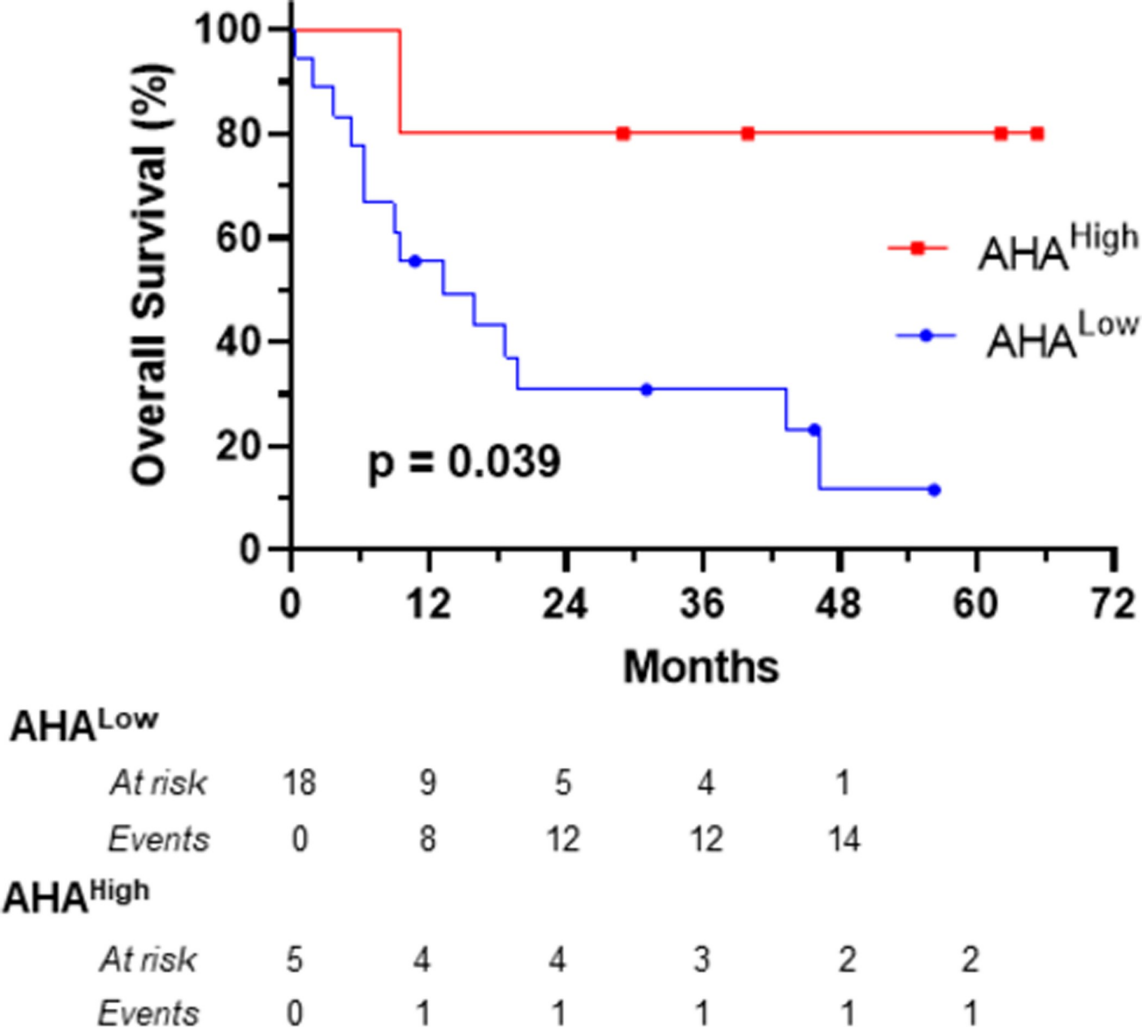
Kaplan-Meier plot of the overall survival of melanoma patients following commencement of treatment with pembrolizumab. Patients were divided, on the basis of their ranked IgG-AHA levels, into AHA^Low^ (n = 18) and AHA^High^ (n = 5) groups using the 75% percentile as a cut-point. Solid symbols on each curve represent patients censored at that specific time. Curves were compared using the log rank test and the p value indicated. Median survival for AHA^Low^ was 13.2 months and for AHA^High^ was not reached.

The distribution of pembrolizumab- F(ab’)_2_ reactive AHA levels in the pembrolizumab treated melanoma patients was positively skewed with a subset having relatively high levels (Fig 1A). The association between high AHA levels and survival was therefore analysed following division of patients into high and low groups using the 75% percentile of AHA levels as a cut-point. The 75% percentile also corresponded to the optimal cut-point determined using maximally selected rank statistics. Patients with high AHA levels had significantly better survival, post commencement of pembrolizumab treatment, than those with lower AHA levels (Fig 3). The clinical characteristics of these groups are described in Table 1. However the low number of patients in the AHA^High^ group (n = 5) precludes meaningful statistical evaluation of whether there are significant differences in the clinical characteristics of these groups.

**Table 1:** Clinical characteristics of pembrolizumab treated patients at time of treatment commencement.

	All	AHA^Low^	AHA^High^
***N***	23	17	5
**Age *(Median*, *range)***	68 (39–91)	67 (47–91)	69 (39–78)
***Male (n*, *%)***	13 (57%)	13 (76%)	2 (40%)
***Liver and/or Brain*** ***Metastasis (N*, *%)***	11 (48%)	9 (53%)	2 (40%)

### Effect of AHA on granulocyte activation

ROS generation is an early marker of granulocyte activation induced by antibody complexes [38]. We therefore investigated whether antibody complexes formed between AHA and F(ab’)_2_ could induce ROS generation.

**Fig 4:**
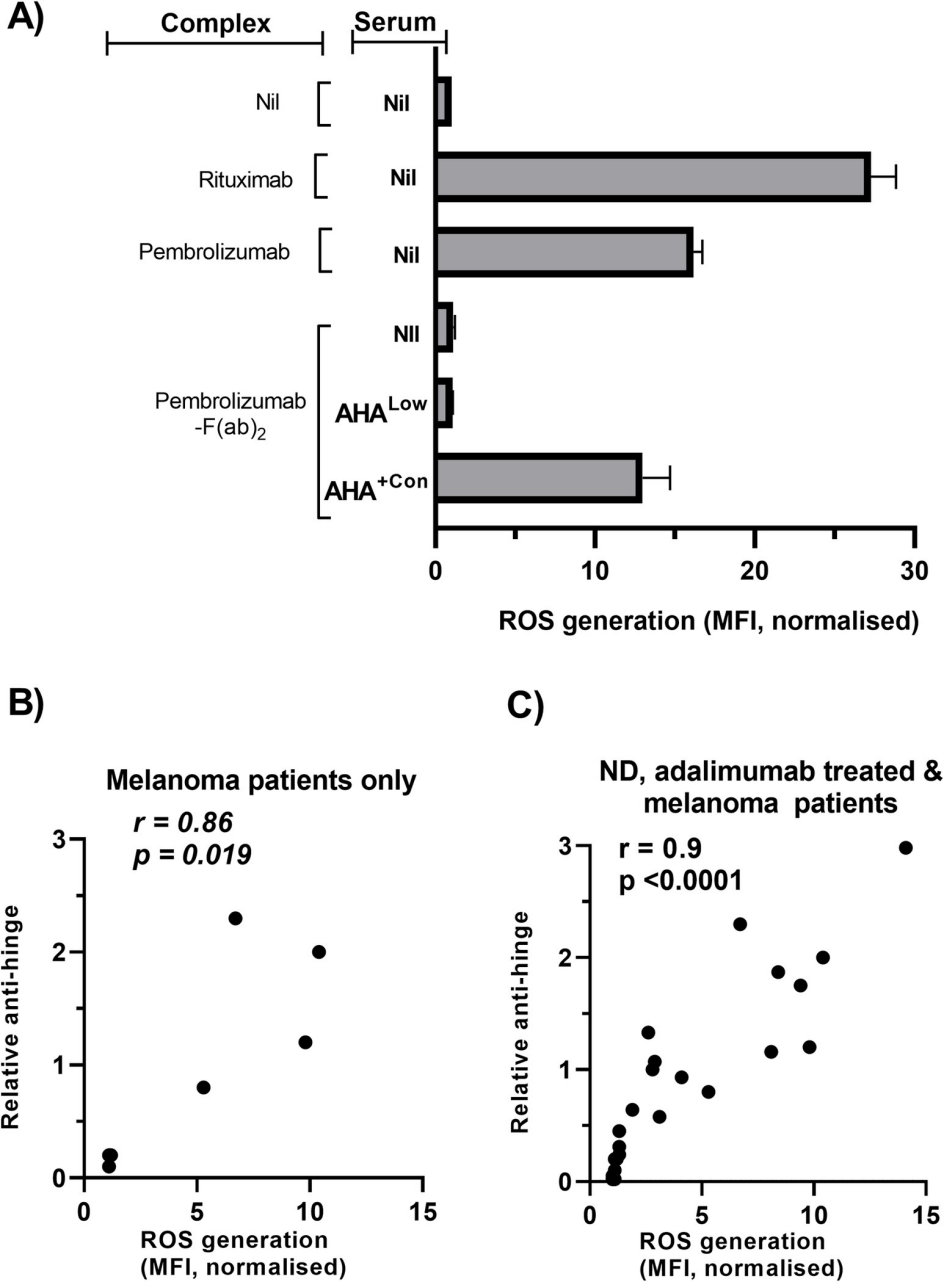
Effect of AHA on ROS production by granulocytes. Granulocytes loaded with ROS indicator (DHR) were added together with serum samples to antibody complexes formed by coating wells with either nil, antibodies (rituximab, pembrolizumab) or pembrolizumab- F(ab´)_2_. Plates were then incubated for 1h prior to flow cytometric analysis (A) Representative bar graph of rhodamine-123 staining observed following culture under the indicated conditions. Data are normalized relative to the MFI of nil treated granulocytes and are from a single experiment of 3 performed. (B, C) ROS stimulating capacity of individual serum samples following addition to cultures containing granulocytes and solid phase pembrolizumab- F(ab´)_2_. Data are shown as a scatterplot of ROS generation versus anti-hinge levels for each individual serum collected from either (B) a mixture of AHA^High^ (n = 4) and AHA^Low^ (n = 3) pembrolizumab treated melanoma patients or (C) normal donors (n = 6), adalimumab treated patients (n = 11) and pembrolizumab treated melanoma patients (n = 7). The spearman rank correlation coefficient is indicated.

Granulocytes cultured alone demonstrated little detectable ROS generation whilst the presence of solid phase pembrolizumab or rituximab strongly induced ROS production (Fig 4A). The presence of solid phase pembrolizumab- F(ab’)_2_ did not induce ROS generation confirming the requirement for signaling via Fc regions. The addition of AHA^low^ serum to cultures containing solid phase pembrolizumab- F(ab’)_2_ similarly did not result in ROS generation. However the presence of both AHA^+Con^ serum and solid phase pembrolizumab-F(ab’)_2_ resulted in increased ROS generation demonstrating that binding of AHA to F(ab’)_2_ provided the Fc regions required for signaling.

Serum from ND and patients were tested for their ability to induce ROS generation in cultures containing granulocytes and solid phase pembrolizumab-F(ab’)_2_ (Fig 4B and 4C). Initial analysis was performed on serum from a subset of pembrolizumab treated patients that included a number of the AHA^Low^ (n = 3) samples and all the AHA^High^ samples (n = 4) (Fig 4B). Those samples showed a clear correlation between AHA levels and the level of ROS generation. The analysis was expanded to include samples from both ND and adalimumab treated patients (Fig 4C). A wide range in the levels of ROS induction was observed and overall there was a strong correlation between AHA levels and ROS generation (r = 0.9, p<0.0001). The addition of serum with elevated AHA in the absence of solid phase F(ab’)_2_ did not induce ROS generation confirming the specificity of this response.

### Effect of AHA on NK activation

Stimulation of NK cells via FcR engagement of appropriate isotypes promotes NK activation in response to IL-2. Such activation is characterised by a reduction in both CD16 expression and cytotoxic activity [8, 39–41]. We therefore investigated whether antibody complexes formed between AHA and F(ab’)_2_ could induce NK activation.

**Fig 5:**
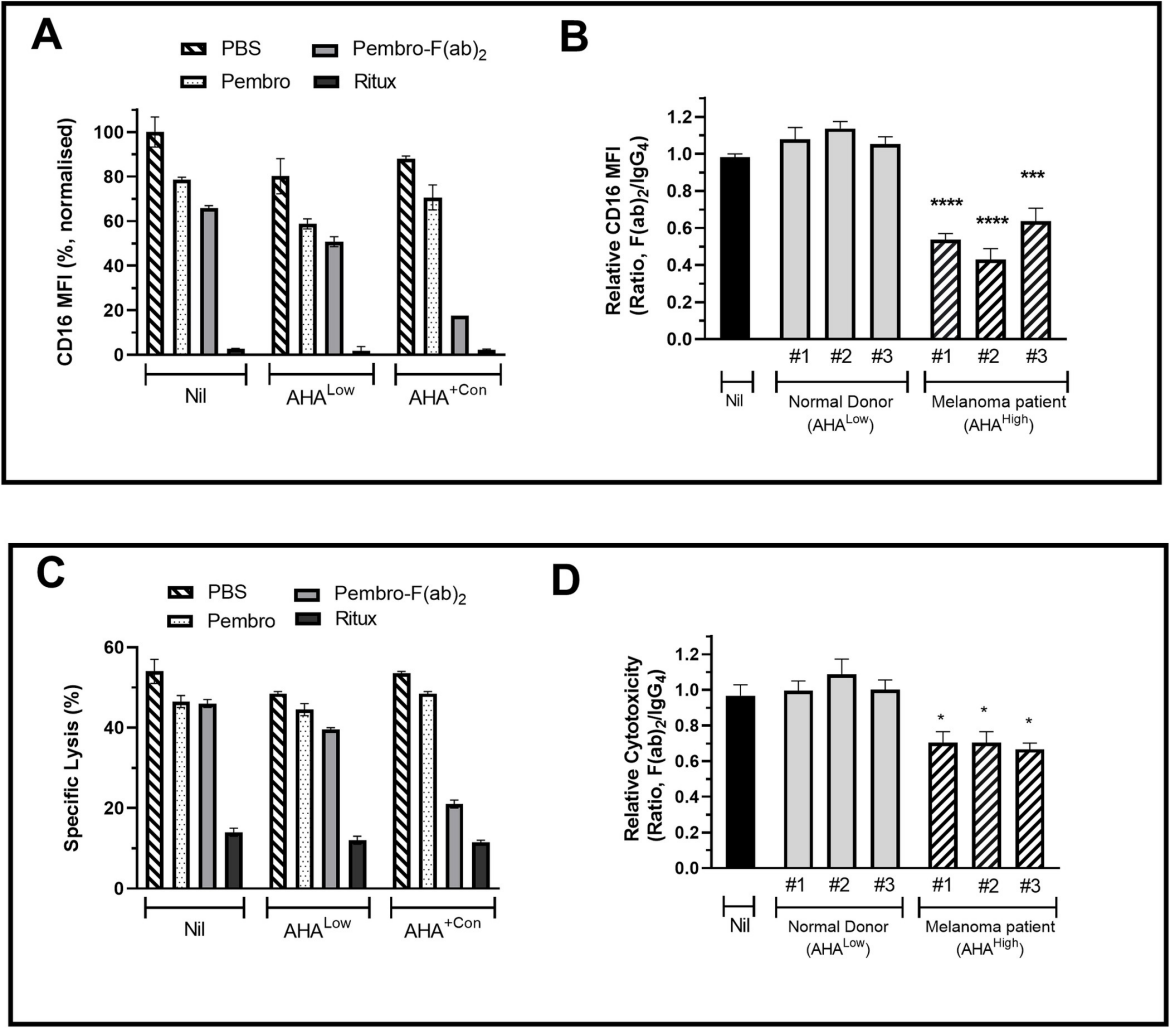
Effect of AHA on NK activation. Responder cells (enriched NK cells or PBMC) and IL-2 were added in combination with test serum samples to wells coated with either nil, antibodies (rituximab, pembrolizumab) or pembrolizumab- F(ab´)_2_. Following 24h incubation, cultures containing (A, B) enriched NK cells were utilised for analysis of CD16 expression (MFI) by flow cytometry and (C, D) PBMC were used as effectors in killing assays utilizing K562 as the target. Data from representative experiments are shown as bar graphs of (A) CD16 expression and (C) Specific cytotoxicity following culture of corresponding responders with the indicated solid phase antibodies in the additional presence of either nil, AHA^Low^ serum or AHA^+Con^ serum (B, D) Bar graphs of the relative levels of CD16 expression and cytotoxicity observed following addition of individual test serum to wells coated with either pembrolizumab or pembrolizumab- F(ab´)_2_. Test sera were from with AHA^Low^ ND or AHA^High^ pembrolizumab treated melanoma patients. For each sera relative levels are defined as the pembrolizumab- F(ab´)_2_/pembrolizumab ratio (F(ab´)_2_/IgG_4_) and pooled data from three separate experiments are shown as mean ± SEM. Statistical analysis was performed using repeated measures ANOVA and asterisks indicate values significantly different from those observed in control wells containing no serum.

NK cells incubated with IL-2 alone had high levels of CD16 expression (Fig 5A) and cytotoxic activity (Fig 5C). The additional presence of solid phase pembrolizumab (IgG_4_) or the corresponding F(ab’)_2_ fragment resulted in small decreases in of these parameters whilst solid phase IgG_1_ antibody (rituximab) induced strong downregulation. A similar pattern was observed when cultures were additionally supplemented with AHA^Low^ serum. In contrast, the presence of AHA^+Con^ serum resulted in strong downregulation in response to solid phase pembrolizumab- F(ab’)_2_ but not pembrolizumab. This suggests that AHA antibodies within serum specifically recognize F(ab’)_2_ and therefore provide Fc regions of the necessary isotypes for NK activation.

Serum from pembrolizumab treated melanoma patients with elevated AHA were therefore tested for their ability to induce downregulation of CD16 and cytotoxic activity in the presence of pembrolizumab- F(ab’)_2_ (Fig 5B and 5D). Serum from ND with low AHA levels was used as a control. The levels observed in the presence of solid phase pembrolizumab was used as a measure of the background response for each sample. The levels observed using solid phase pembrolizumab- F(ab’)_2_ were used as a measure of the AHA derived response and the ratio of F(ab’)_2_/IgG_4_ signals was used as a measure of the relative response for each sample. Relative to the responses observed in the presence of solid phase pembrolizumab, none of the tested AHA^Low^ serums had a significant effect on responses to pembrolizumab- F(ab’)_2_. Sera from all three pembrolizumab treated melanoma patients induced significant downregulation of CD16 and cytotoxic activity in response to pembrolizumab- F(ab’)_2_. As a further check on the specificity of this effect a PD-1^+^ cell line was labelled with either nil, pembrolizumab or pembrolizumab- F(ab’)_2_ then used to deplete AHA^+^ sera prior to analysis in the NK activation assay (S2 Fig). The AHA^+^ sera alone induced strong downregulation of CD16. Pre-depletion of the serum with either unlabelled or pembrolizumab labelled cells did not modulate the downregulation. However depletion with pembrolizumab- F(ab’)_2_ labelled cells resulted in almost complete reversal of this effect.

### Effect of AHA on CDC and C3b deposition

**Fig 6:**
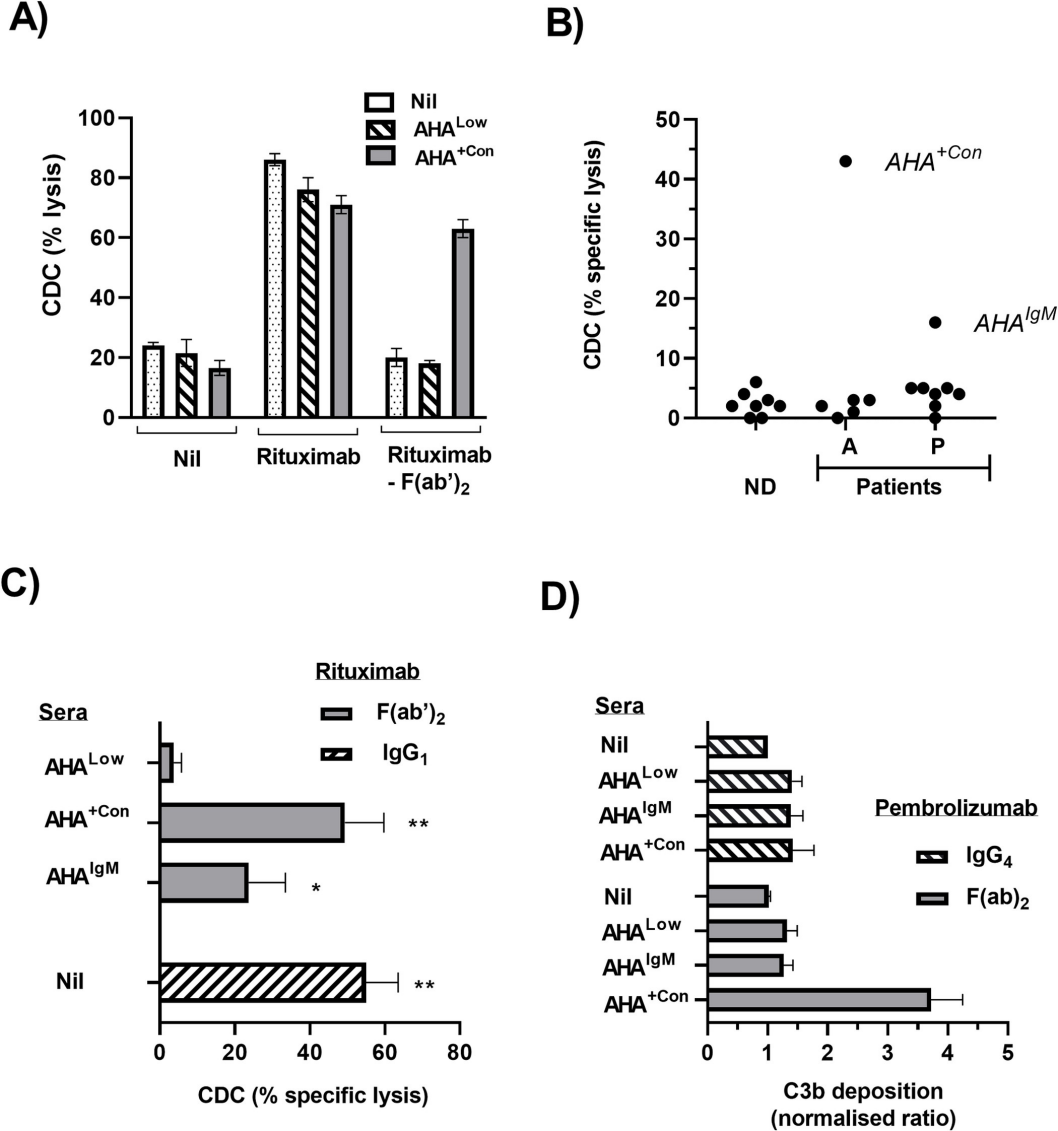
Effect of AHA on CDC and C3b deposition. (A, B) CDC of Raji, a CD20^+^ cell line. Raji was incubated with nil, Rituximab or rituximab- F(ab´)_2_ either alone or in combination with ND or patient serum. Following subsequent exposure to complement the level of target cell lysis (%) was determined. (A) Data from a representative experiment of three performed are shown as a bar graph of % lysis observed following incubation of Raji with the indicated combinations of antibody (rituximab vs rituximab- F(ab´)_2_) and either nil AHA^Low^ or AHA^+Con^ sera (B) Serum samples from ND, adalimumab treated patients [A] and pembrolizumab treated melanoma patients [P] were added to Raji in both the presence and absence of rituximab- F(ab´)_2_. The difference between the treatments following exposure to complement provided the measure of specific lysis and data are shown as a scatter plot (C) CDC inducing activity of serum from a pembrolizumab treated melanoma patient (AHA^IgM^) compared to a ND with low AHA levels (AHA^Low^) and AHA^+Con^. Sera were combined with rituximab- F(ab´)_2_ or nil and their ability to induce specific lysis of Raji was determined in comparison to that induced by rituximab alone. Pooled data from three separate experiments are shown as mean ± SEM. Statistical analysis was performed using a repeated measures ANOVA in combination with a Holm-Šídák’s multiple comparisons test and asterisks indicate values significantly different from those observed AHA^Low^ sera. (D) Effect of AHA on C3b deposition. Wells coated with solid phase pembrolizumab or pembrolizumab- F(ab´)_2_ were incubated with nil sera, AHA^Low^ sera, AHA^+Con^ or AHA^IgM^ sera and then following exposure to complement the level of C3b deposition was determined by ELISA. Data from individual experiments (*n* = 3) were normalized relative to the OD observed in control wells coated with pembrolizumab + nil serum (defined as ratio = 1). Pooled data are shown as mean ± SEM and asterisks indicate values significantly different from the control following analysis using a repeated measures ANOVA.

The CD20 mAb Rituximab has a well characterized ability to induce CDC of CD20^+^ B cell lines such as Raji. In the absence of rituximab only low levels of cell death were observed whilst addition of rituximab resulted in high levels of cell death in Raji irrespective of whether nil, AHA^Low^ or AHA^+Con^ serum were also present (Fig 6A). In contrast, addition of Rituximab- F(ab’)_2_ did not result in increased Raji lysis in cultures containing either nil or AHA^Low^ serum.

However high levels of Raji death were restored when cultures contained both rituximab-F(ab’)_2_ and AHA^+Con^ serum.

All serum with elevated AHA were screened for their ability to induce CDC function by adding them to Raji in combination with either nil or rituximab-F(ab’)_2_ fragments and then determining the specific rituximab-F(ab’)_2_ targeted lysis (Fig 6B). The only serum from adalimumab treated patients able to induce CDC was one with the highest AHA levels, which was also used as the positive control (AHA^+Con^) throughout the study. Only one of the sera from pembrolizumab treated melanoma patients sera demonstrated CDC activity in initial screening and this serum (AHA^IgM^) was distinguished by having IgM-AHA but no IgG-AHA. Further analysis using those 2 serums in combination with Rituximab- F(ab’)_2_ confirmed that presence AHA^+Con^ induced similar levels of CDC to that induced by rituximab alone whilst AHA^IgM^ induced significant but lower levels of CDC (Fig 6C). Both AHA^+Con^ and AHA^IgM^ were also tested for their ability to induce C3b deposition on solid phase pembrolizumab- F(ab’)_2_ (Fig 6D). Only the adalimumab patient sera AHA^+Con^ was able to induce significant increases in C3b deposition.

## Discussion

The proteolytic cleavage of antibodies in their hinge region generates cryptic epitopes that maybe recognised by circulating AHA. The levels and potential functional implications of AHA in patients receiving the PD-1 specific monoclonal pembrolizumab were therefore investigated.

It is well established that AHA can be detected in normal human serum and that the incidence and levels of AHA are elevated in patients with chronic inflammatory disorders [9, 10, 12–14]. The results of the current study are consistent with those reports in that AHA levels were elevated in patients receiving adalimumab as a treatment for inflammatory conditions. The wide range of levels observed in those patients was also a feature of previous studies and thought to reflect differences in disease activity.

Cancer development and progression is also closely associated with both tumour localized and/or systemic inflammation [42]. However, AHA levels in cancer patients, or indeed any patient group receiving therapeutic antibodies, has not been previously reported. To date, analysis of AHA reactivity with IgG_4_ antibodies has been restricted to studies using either the therapeutic monoclonal Natalizumab [13, 14] or chimeric anti-biotin monoclonals [12] which lack the hinge stabilizing S228P mutation present in pembrolizumab and nivolumab. Conformational differences between the F(ab’)_2_ fragments of natalizumab, pembrolizumab and nivolumab have been reported [15, 30] and, as some AHA recognise conformational epitopes, it cannot be assumed AHA will react with all IgG_4_ antibodies. However, the results of the current study demonstrate that pembrolizumab, and to a lesser extent nivolumab, can be recognized by AHA. Analysis of sera collected at different time points (Fig 1F) also indicates that in pembrolizumab treated patients elevated AHA can be present prior to treatment and that levels remain relatively stable during treatment. This continued presence of elevated AHA levels has not been documented before and contrasts with the often transient development of anti-drug antibodies observed in patients receiving therapeutic antibodies [43]. In the current study, overall levels of IgG-AHA in pembrolizumab treated patients were not as high as those observed in adalimumab treated patients. However, elevated IgG-AHA levels were detected in a subset of these patients. The elevated reactivity was directed against F(ab’)_2_ fragments of both IgG_4_ (pembrolizumab, nivolumab) and IgG_1_ (adalimumab, rituximab) therapeutic monoclonals in 4 patients and a further two patients had elevated reactivity against the IgG1- F(ab’)_2_ but not IgG4- F(ab’)_2_. The finding that sera reactivity with cleaved IgG_1_ is more widespread than reactivity with cleaved IgG_4_ is consistent with previous studies of inflammatory disease patients [12]. The finding that individual sera can contain AHA reactive with F(ab’)_2_ derived from both IgG_1_ and IgG_4_ may appear surprising given these isotypes have some differences in their hinge region amino acid sequences and the C-terminal amino acid exposed after pepsin cleavage also differs [12]. However although these c-terminal amino acids are different they are both large hydrophobic AA and it has been demonstrated that some monoclonal AHA can cross-react with hinge regions that terminate with non-identical but structurally similar amino acids [15]. Furthermore current data indicates that in the majority of patients with elevated AHA the AHA repertoire is polyclonal and recognizes a range of the F(ab’)_2_ fragments that can be derived from different isotypes using different proteases [14, 15]. Therefore sera may contain a mix of individual AHA that each recognise F(ab’)_2_ derived from only one isotype in addition to AHA that cross-react.

The specificity of the AHA ELISA was confirmed by both inhibition and depletion experiments. These experiments also provided strong evidence that the ELISA detects AHA reactive with the epitopes accessible on antigen bound forms of F(ab’)_2_ fragments and suggests at least some of these epitopes are less accessible on the soluble forms of F(ab’)_2_ fragments. Additionally these experiments provided strong evidence that Fc reactive antibodies such as rheumatoid factors (RF) do not contribute to the ELISA signal. Further evidence that RF were not a potential source of false positive signals in the AHA ELISA results was provided by the observations that (i) only one of the tested sera had detectable IgM-RF present and (ii) that the target F(ab’)_2_ fragments were not recognised by the anti-human Fc reagent used in the AHA ELISA, thereby demonstrating that there were no Fc regions present which could provide a target for RF binding.

The immediate functional consequence of hinge cleavage is the generation of fragments that retain antigen binding capacity but lack Fc effector function. Studies to date have clearly demonstrated that anti-hinge antibodies can restore ADCC and CDC function to cleaved monoclonals. However, those studies predominantly utilised monoclonal AHA or AHA purified from pooled serum, which preferentially enriches those of highest affinity [9, 15, 16, 20, 23]. Present data indicates the AHA repertoire differs between individuals and is comprised of multiple isotypes, specificities and affinities [14, 15]. Given that the functional effect of specific antibodies in serum can be modulated by the presence of other antibodies directed against the same antigen [32, 33] it is unclear what level of AHA mediated functional effects will be observed in the more physiological setting of individual sera that contains a full repertoire of AHA. To date only a single study has utilised serum samples and reported that the AHA repertoire in 2 individual serums was capable of restoring complement activation activity to F(ab’)_2_ fragments [13].

Although not able to induce CDC or ADCC, the Fc region of IgG_4_ mAbs such as pembrolizumab can induce neutrophil activation [8, 44]. In the current study we demonstrate that AHA^+^ sera can restore this ability to cleaved pembrolizumab and that the level of AHA detected in individual sera strongly correlates with their neutrophil activation capacity. As neutrophil activation can be induced by a range of isotypes, neutrophil activation is likely to reflect total AHA binding.

The Fc mediated stimulation of NK activation is more isotype restricted due to the relatively low affinity of the primary Fc receptor of NK cells (CD16a) for IgG_2_ and IgG_4_ antibodies [45]. Consequently, pembrolizumab has limited ability to induce NK activation. Analysis of sera from three pembrolizumab treated patients with high AHA levels demonstrated that all could, through binding to pembrolizumab- F(ab’)_2,_ provide Fc isotypes capable of inducing NK activation. As NK cells play an important role in cancer immune surveillance this activation has the potential to induce anti-tumour responses distinct from those resulting from PD-1 blockade alone [46]. In this regard, it is notable that a recent study using *in vivo* tumour models reported that NK cells played a major role in the anti-tumour effects observed following AHA administration [22]. The induction of CDC by antibodies is similarly isotype dependent but FcR independent. The killing of B cells labelled with CD20 mAbs such as rituximab is the best characterised example of CDC. Screening of all patient sera with moderate-high AHA levels identified only two individual sera with the ability to provide surrogate CDC capacity to rituximab- F(ab’)_2_ fragments. One of these was from an adalimumab treated patient and had the highest IgG-AHA levels detected in the study. The other was from a pembrolizumab treated patient and the only detectable AHA in the serum were of the IgM class. Therefore, its activity may reflect the higher CDC activity of IgM versus IgG [47]. The sera with the highest levels of IgG-AHA was capable of inducing both CDC and NK activation. However, none of the three sera from pembrolizumab treated patients shown to be capable of inducing NK activation were also able to induce CDC. This likely reflects the stringent spatial requirements for antibodies to induce CDC [48]. In addition the binding of non-complement fixing AHA isotypes may inhibit the activity of any complement fixing AHA that have also bound [49]. Therefore, given that the repertoire of AHA in individual sera may include multiple specificities and isotypes [14, 15] it is not surprising that the ability to induce CDC was more restricted than FcR stimulating ability. This difference was not apparent in previous AHA studies where functional effects were evaluated using purified high affinity or monoclonal AHA. Therefore, the potential importance of CDC in the context of cleaved pembrolizumab and AHA is unclear.

An association between AHA levels and survival in cancer patients has not been previously reported. The detection of cleaved IgG at tumour sites has however been associated with both tumour progression and poor clinical outcomes [18, 21, 22]. Studies using murine tumour models have also demonstrated that administration of AHA can reverse these effects [22, 24]. The finding, in the current study, that elevated AHA levels are associated with increased survival also supports the possibility that AHA may increase anti-tumour immunity. However, the low number of patients analysed represents a major limitation and this data must be interpreted with considerable caution. A larger study is required to confirm these results and such a study would also allow analysis of whether AHA represent an independent predictor of survival or are merely a surrogate marker of other clinical or known prognostic factors. Although melanoma progression is associated with the presence of a number of proteases capable of antibody cleavage [25–28], and pembrolizumab is sensitive to proteolysis [19], it is currently unknown whether such cleavage actively occurs *in vivo*. Therefore, although it is clear that AHA can modulate immune responses in some settings, the possibility that AHA have limited functional impact in melanoma patients and are primarily a marker of a more activated or primed immune system cannot be discounted.

A potential limitation of the current study is that only pepsin generated F(ab’)_2_ were used to detect AHA. Both IgG_1_ and IgG_4_ are cleaved within the lower hinge by a number of inflammatory and/or tumor associated proteases -MMP-3 and MMP-12 at one site, Cathespin at a second site and by MMP-7/pepsin at a third site [11]. AHA reactive with all 3 sites have been reported and AHA recognising MMP-7/pepsin cleaved IgG_4_ have been reported to provide the most discriminatory disease marker, at least in RA patients [12–14]. Current data indicates that in the majority of patients with elevated AHA the AHA repertoire is polyclonal and recognizes a range of F(ab’)_2_ fragments [14, 15]. This suggests that in the absence of any data on which specific protease(s) are the most relevant in the pembrolizumab treated melanoma, pepsin generated F(ab’)_2_ can be used as a broad marker of AHA generation. It must however be acknowledged that functional activities that are acutely sensitive to epitope location and/or orientation, such as CDC, may only be observed using AHA in combination with antibody fragments derived using a specific protease [15].

In conclusion, this study provides the first demonstration, in any cancer type, that AHA can be detected at elevated levels in at least a subset of patients. The detection of AHA in melanoma patients being treated with a therapeutic anti-PD-1 monoclonal raises the possibility they may have a functional impact in a setting where tumour and/or inflammation driven cleavage of the monoclonal occurs. In the current study cleaved pembrolizumab was, as expected, unable to induce Fc mediated responses. However, the presence of AHA not only provided F(ab’)_2_ -pembrolizumab with the same Fc stimulating capacity as pembrolizumab but additionally provided the ability to induce NK activation and in some instances CDC. The issue of whether tumour associated proteolysis of PD-1 mAbs and subsequent AHA recognition impacts on treatment efficacy requires further study as does the potential prognostic value of AHA levels.

## Supporting information

S1 Fig Detection of IgA and IgM antibodies reactive with F(ab’)_2_ or IgG_4_. Serum from normal donors [ND], adalimumab treated patients [A] and pembrolizumab treated patients [P] were analysed by ELISA for the presence of IgA and IgM antibodies reactive with either pembrolizumab-F(ab’)_2_ fragments or pembrolizumab. (A, B) Scatter plot of reactivity with pembrolizumab-F(ab’)_2_ fragments observed following either (A) IgA or (B) IgM specific detection of bound antibodies. (C) Scatter plot of reactivity with pembrolizumab observed following IgM specific detection of bound antibodies. Data are shown as O.D’s and are from a representative experiment of 2 performed using each ELISA.
(TIF)

S2 Fig Effect of AHA depletion on NK activation. Responder cells (enriched NK cells) and IL-2 were added in combination with test samples to wells coated with either pembrolizumab or pembrolizumab- F(ab´)_2_. Following 24h incubation, cultures were utilised for analysis of CD16 expression (MFI) by flow cytometry. Test samples were either ND serum (AHA^Low^) alone or a serum from a pembrolizumab treated melanoma patient (AHA^High^) that had been immunodepleted by incubation with either nil or a PD-1^+^ cell line that had been pre-labelled with either nil, pembrolizumab or pembrolizumab- F(ab’)_2_. For each test sample relative levels are defined as the pembrolizumab- F(ab´)_2_/pembrolizumab ratio (F(ab´)_2_/IgG_4_) of CD16 MFI. Data are shown as a bar graph of relative CD16 expression and are from a representative experiment of two performed.
(TIF)

S1 Dataset Data used to build Fig 1 graphs. (XLSX)

S2 Dataset Data used to build Fig 2 graphs. (XLSX)

S3 Dataset Data used to build Fig 3 graph. (XLSX)

S4 Dataset Data used to build Fig 4 graphs. (XLSX)

S5 Dataset Data used to build Fig 5 graphs. (XLSX)

S6 Dataset Data used to build Fig 6 graphs. (XLSX)
