# Supplementary figures and images for: Levels and in vitro functional effects of circulating anti-hinge antibodies in melanoma patients receiving the immune checkpoint inhibitor pembrolizumab
Source: PLoS One. 2023 Sep 15;18(9):e0290793. doi: 10.1371/journal.pone.0290793 (PMC10503750; doi:10.1371/journal.pone.0290793)

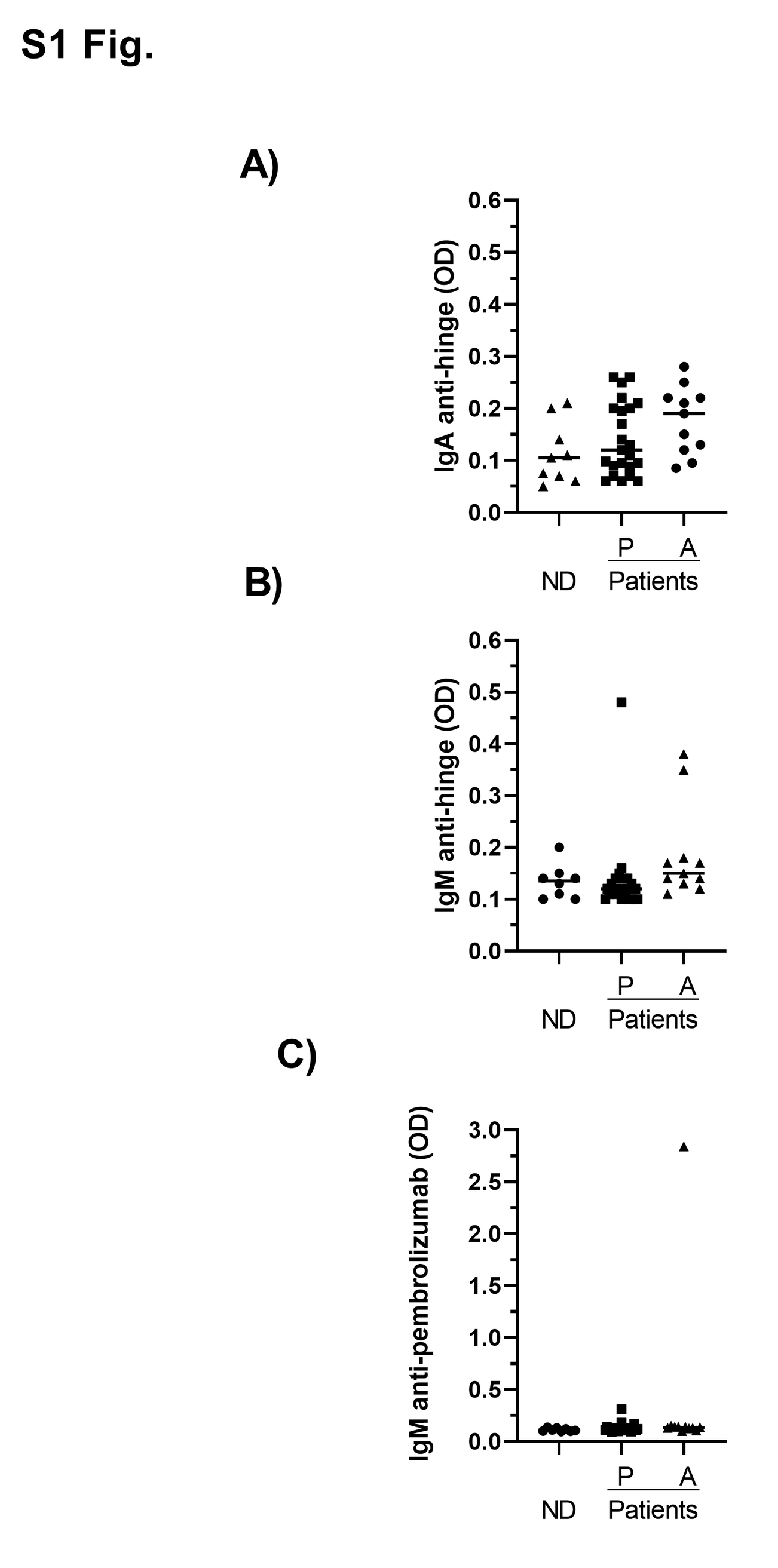

Supplement: S1 Fig — Serum from normal donors [ND], adalimumab treated patients [A] and pembrolizumab treated patients [P] were analysed by ELISA for the presence of IgA and IgM antibodies reactive with either pembrolizumab-F(ab’)2 fragments or pembrolizumab. (A, B) Scatter plot of reactivity with pembrolizumab-F(ab’)2 fragments observed following either (A) IgA or (B) IgM specific detection of bound antibodies. (C) Scatter plot of reactivity with pembrolizumab observed following IgM specific detection of bound antibodies. Data are shown as O.D’s and are from a representative experiment of 2 performed using each ELISA. (TIF) [file pone.0290793.s001.tif]

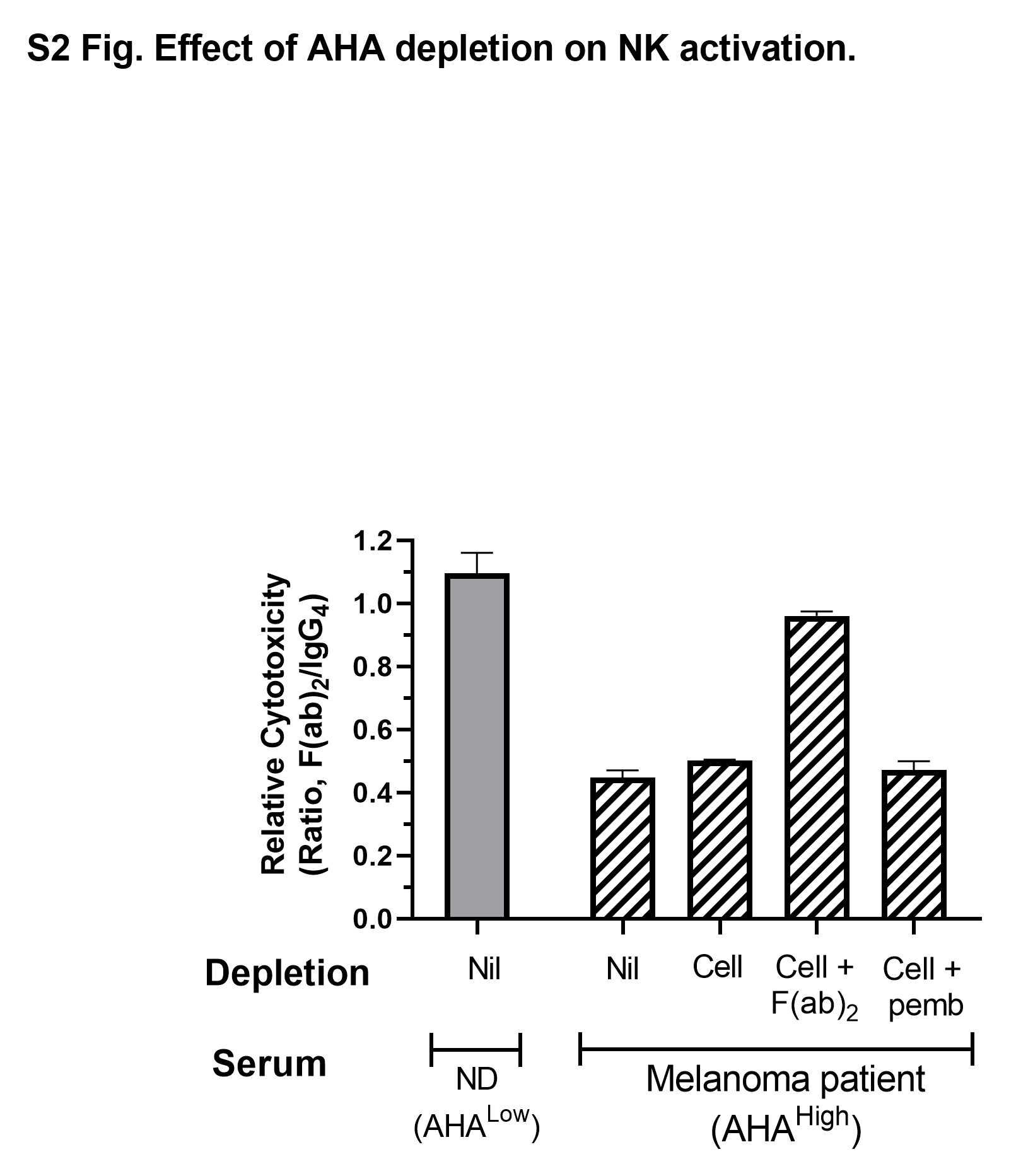

Supplement: S2 Fig — Responder cells (enriched NK cells) and IL-2 were added in combination with test samples to wells coated with either pembrolizumab or pembrolizumab- F(ab´)2. Following 24h incubation, cultures were utilised for analysis of CD16 expression (MFI) by flow cytometry. Test samples were either ND serum (AHALow) alone or a serum from a pembrolizumab treated melanoma patient (AHAHigh) that had been immunodepleted by incubation with either nil or a PD-1+ cell line that had been pre-labelled with either nil, pembrolizumab or pembrolizumab- F(ab’)2. For each test sample relative levels are defined as the pembrolizumab- F(ab´)2/pembrolizumab ratio (F(ab´)2/IgG4) of CD16 MFI. Data are shown as a bar graph of relative CD16 expression and are from a representative experiment of two performed. (TIF) [file pone.0290793.s002.tif]
